# Fbxw7 is an independent prognostic marker and induces apoptosis and growth arrest by regulating YAP abundance in hepatocellular carcinoma

**DOI:** 10.1186/1476-4598-13-110

**Published:** 2014-05-17

**Authors:** Kangsheng Tu, Wei Yang, Chao Li, Xin Zheng, Zhongtang Lu, Cheng Guo, Yingmin Yao, Qingguang Liu

**Affiliations:** 1 Department of Hepatobiliary Surgery, the First Affiliated Hospital of Xi’an Jiaotong University, Xi’an, Shaanxi 710061, China

**Keywords:** Fbxw7, Hepatocellular carcinoma, Hippo-YAP pathway, Apoptosis, Growth arrest

## Abstract

**Background:**

The E3 ubiquitin ligase Fbxw7 functions as a general tumor suppressor by targeting several well-known oncoproteins for ubiquitination and proteasomal degradation. However, the clinical significance of Fbxw7 and the mechanisms involved in the anti-cancer effect of Fbxw7 in HCC are not clear.

**Method:**

The Fbxw7 and YAP expression in 60 samples of surgical resected HCC and matched normal tumor-adjacent tissues were assessed using IHC or immunoblotting. Flow cytometry, caspase 3/7 activity assay, BrdU cell proliferation assay and MTT assay were used to detect proliferation and apoptosis of HCC cells. The regulatory effect of Fbxw7 on YAP in HCC cells was confirmed by qRT-PCR, immunoblotting and immunofluorescence. Co-immunoprecipitation was used to analyze interaction between YAP and Fbxw7. Nude mice subcutaneous injection, Ki-67 staining and TUNEL assay were used to evaluate tumor growth and apoptosis *in vivo*.

**Results:**

In this study, we found that Fbxw7 expression was impaired in HCC tissues and loss of Fbxw7 expression was correlated with poor clinicopathological features including large tumor size, venous infiltration, high pathological grading and advanced TNM stage. Additionally, we demonstrated that patients with positive Fbxw7 expression had a better 5-year survival and Fbxw7 was an independent factor for predicting the prognosis of HCC patients. We confirmed that Fbxw7 inhibited HCC by inducing both apoptosis and growth arrest. Elevated YAP expression was observed in the same cohort of HCC tissues. Pearson's correlation coefficient analysis indicated that Fbxw7 was inversely associated with YAP protein expression in HCC tissues. We also found that Fbxw7 regulated YAP protein abundance by targeting YAP for ubiquitination and proteasomal degradation in HCC. Furthermore, restoring YAP expression partially abrogated Fbxw7 induced HCC cell apoptosis and growth arrest *in vitro* and *in vivo*.

**Conclusion:**

These results indicate that Fbxw7 may serve as a prognostic marker and that YAP may be a potential target of Fbxw7 in HCC.

## Introduction

Fbxw7 (F-box and WD repeat domain-containing 7) also known as Fbw7, a well-known F-box protein in the SCF (SKP1-CUL1-F-box protein) E3 ligase complex, determines target specificity by recognizing and binding proteins, leading to their ubiquitination and proteasomal degradation [1]. Fbxw7 has been characterized as a general tumor suppressor in human cancer and plays a critical role in cell cycle progression, apoptosis, tumor metastasis and drug resistance [1]. Recent studies have identified several specific substrates of Fbxw7 including c-Myc [2], Cyclin E [3], c-Jun [4], SREBP1 (Sterol regulatory element binding protein-1) [5], mTOR (Mammalian target of rapamycin) [6], Notch-1 [7] and MCL-1 (Myeloid cell leukemia-1) [8]. These target proteins typically contain the conserved CPD (Cdc4 phospho-degron) (L)-X-pT/pS-P-(P)-X-pS/pT/E/D (X = any amino acid) motif and the amino residues in CPD must first be phosphorylated in order for Fbxw7 to efficiently recognize and mediate the ubiquitination of the target protein [1,9].

Reduced Fbxw7 expression is often observed in multiple human cancers including breast cancer, colorectal cancer, gastric cancer, prostate cancer, pancreatic cancer and HCC (Hepatocellular carcinoma) [10]. Akhoondi et al. performed a comprehensive genetic screen of primary tumors. Their data showed that gene mutation leads to Fbxw7 inactivation in various types of human cancers, and overall mutation frequency was approximately 6% [11]. Yakobori et al. shown that low Fbxw7 expression was significantly correlated with lymph node metastasis, tumor size and poor prognosis in gastric cancer [12]. Loss-of-function mutations of Fbxw7 led to the accumulation of Cyclin E and played a key role in the progression of human pancreatic cancer [13]. A recent study reported that Fbxw7 inhibited melanoma cell migration and served as a prognostic marker [14]. Our previous studies have demonstrated that low Fbxw7 expression contributes to the more aggressive phenotypes seen in HCC; Fbxw7 knockdown promoted tumor cell proliferation and decreased p53-induced apoptosis *in vitro*[15-17]. However, the clinical significance of Fbxw7 in predicting prognosis and the mechanisms involved in the anti-cancer effects of Fbxw7 are unknown.

The Hippo signaling pathway, a well-conserved potent regulator of cell growth and apoptosis in mammals, was initially discovered in *Drosophila*[18]. YAP (Yes-associated protein), a negatively regulated downstream effector of the Hippo pathway, functions as a transcriptional coactivator, which interacts with several transcription factors including ErbB4, RUNX2 (Runt-related transcription factor 2), p73 and TEAD (TEA domain) transcription factor family members [19,20]. YAP overexpression and nuclear accumulation has been reported in various types of human cancers, including colorectal cancer, ovarian cancer, lung cancer and HCC [21]. YAP was identified as the driving oncogene of the 11q22 amplicon seen in HCC and breast cancer [22,23]. Transgenic mice with liver-targeted YAP overexpression resulted in hepatomegaly and eventually developed liver tumors or severe dysplasia [24]. In clinical studies, YAP was an independent factor in predicting a poor disease-free survival and overall survival in HCC [25]. Several lines of evidence from *in vitro* and *in vivo* studies have suggested that YAP plays a critical role in hepatocarcinogenesis. A recent study reported that a β-TrCP E3 ubiquitin ligase regulated YAP stability through ubiquitination and proteasomal degradation [26]. However, otherwise the mechanisms governing YAP protein stability remain poorly understood.

In this study, we demonstrate that Fbxw7 is an independent prognostic factor for predicting both the overall and the disease-free 5-year survival of HCC patients. Fbxw7 acted as a tumor suppressor by promoting both apoptosis and growth arrest in HCC. Fbxw7 was inversely related to YAP protein expression in HCC tissues. Interestingly, Fbxw7 interacted with YAP and catalyzed YAP ubiquitination, ultimately leading to YAP degradation. Importantly, the anti-cancer effect of Fbxw7 could be partially inverted by restoring YAP expression *in vitro* and *in vivo*. Our results suggest that Fbxw7 may target YAP for ubiquitination and proteasomal degradation, thereby inhibiting HCC growth and hence tumor progression.

## Materials and methods

### Clinical samples, cell lines and expression vectors

Sixty HCC samples and paired normal tumor-adjacent samples (>2 cm distance from the margin of the resection) were obtained during surgery and used after obtaining informed consent. All patients underwent the resection of their primary HCC in the Department of Hepatobiliary Surgery at the First Affiliated Hospital of Xi’an Jiaotong University between 2006 and 2008, with a median follow-up time of 32.5 months. The demographic features and clinicopathologic data are shown in Table 1. In summary, the median age of the patients was 52 years (range, 24–72 years). Tumor tissue and matched normal tumor-adjacent tissue specimens were collected and immediately stored in paraformaldehyde for immunohistochemistry. The Xi’an Jiaotong University Ethics Committee approved all protocols according to the 1975 Helsinki Declaration.

**Table 1:** Clinical correlation of Fbxw7 expression in HCC

**Clinicopathologic features**		**Total no. of patients, n = 60**	**No. of patients**		***P***
			**Fbxw7 negative**	**Fbxw7 positive**	
Age (y)	<50	19	13	6	0.365
	≥50	41	23	18	
Sex	Male	48	28	20	0.598
	Female	12	8	4	
HBV	Absent	14	9	5	0.709
	Present	46	27	19	
Serum AFP level (ng/mL)	<400	24	14	10	0.830
	≥400	36	22	14	
Tumor size (cm)	<5	21	7	14	0.002*
	≥5	39	29	10	
No. of tumor nodules	1	45	24	21	0.068
	≥2	15	12	3	
Cirrhosis	Absent	28	17	11	0.916
	Present	32	19	13	
Venous infiltration	Absent	31	14	17	0.015*
	Present	29	22	7	
Edmondson-Steiner grading	I + II	18	6	12	0.006*
	III + IV	42	30	12	
TNM tumor stage	I + II	40	19	21	0.005*
	III + IV	20	17	3	

HCC, hepatocellular carcinoma; HBV, hepatitis B virus; AFP, alpha-fetoprotein; TNM, tumor-node-metastasis. *Statistically significant.

Human HCC cell lines HepG2 and Hep3B, and 293 T and HEK293 cells were obtained from the Institute of Biochemistry and Cell Biology, Chinese Academy of Sciences (Shanghai, China). All the cells were maintained in Dulbecco’s modified Eagle medium (DMEM, Gibco, Grand Island, NY, USA) containing 10% fetal bovine serum (FBS, Gibco) with 100 units/mL penicillin and 100 μg/mL streptomycin (Sigma, St-Louis, MO, USA) and cultured in a humidified 5% CO2 incubator at 37°C.

The pRetrosuper Fbxw7 shRNA (Plasmid #15660) was obtained from Addgene [27]. Retroviral vectors pMMP-Flag-Fbxw7 and pMMP-HA-YAP were generated by inserting the respective cDNA into pMMP. All constructs were confirmed by sequencing and WB analysis. The day before transfection, 5 × 10^6^ 293 T cells were seeded in 100 mm dishes. Three plasmids, 1.5 μg pMD. MLV, 0.5 μg pVSV.G and 2 μg relevant retroviral vectors, were transfected into cells by using Effectene transfection reagent (Qiagen, Valencia, CA, USA). The medium containing the retroviruses was collected 48 and 72 hours after transfection. Viral transduction was done by incubating cells with the viral supernatant (25%) supplemented with polybrene (8 μg/mL) overnight at 37°C. Further experiments were performed 48–96 hours after viral transduction.

### Immunohistochemical staining

Immunohistochemistry was performed on paraformaldehyde-fixed paraffin sections. The following antibodies were used in immunohistochemistry along with a streptavidin peroxidase conjugate (SP-IHC): Fbxw7 (WH0055294M2, Sigma) (1:100), YAP (PA1-46189, Thermo Scientific, Rockford, IL, USA) (1:100) and Ki-67 (#9027, Cell Signaling, Danvers, MA, USA) (1:400). Immunohistochemistry was performed as previous reported [15]. The percentage of positive tumor cells or hepatocytes was graded as per the following criteria: 0, less than 10%; 1, 10–30%; 2, 30–50%; 3, more than 50% [25].

### Real-time quantitative reverse transcription polymerase chain reaction (qRT-PCR)

The following primers were used: YAP sense primer 5’-CCTGCGTAGCCAGTTACCAA-3’ and antisense primer 5’-CCATCTCATCCACACTGTTC-3’ and 18S sense primer 5’-AAACGGCTACCACATCCAAG-3’ and antisense primer 5’-CCTCCAATGGATCCTCGTTA-3’. The PCR amplification for the quantification of the Fbxw7 and YAP mRNAs and the 18S rRNA was performed using an ABI PRISM 7300 Sequence Detection System (Applied Biosystems, Foster City, CA, USA) and a SYBR® Premix Ex Taq™ ii (Perfect Real Time) Kit (Takara Bio, Shiga, Japan), as previous reported [17].

### Immunoblotting

The following primary antibodies were used in the Western blot assays: Fbxw7 (1:1000), YAP (1:1000), Ub (sc-8017, Santa Cruz, CA, USA) (1:500) and β-Actin (sc-47778, Santa Cruz) (1:1000). Horseradish peroxidase-conjugated goat anti-mouse or anti-rabbit secondary antibodies (Bio-Rad, Hercules, CA, USA) were used at a 1:1000–1:5000 dilution and detected using a Western Blotting Luminol Reagent (sc-2048, Santa Cruz).

### Immunofluorescence (IF)

HCC cells were fixed with 3% paraformaldehyde and permeabilized using 0.2% Triton X-100. Then the fixed cells were incubated with the YAP (1:500) primary antibody. The secondary antibody is an Alexa Fluor–conjugated IgG (Invitrogen, Carlsbad, CA, USA). Fluorescence confocal images were captured using a LSM 5 Pascal Laser Scanning Microscope (Zeiss Germany, Oberkochen, Germany) using a × 40 lens and Laser Scanning Microscope LSM PASCAL software (version 4.2 SP1).

### Co-immunoprecipitation (co-IP)

HA (12CA5, Roche, Indianapolis, IN, USA) and Flag (F1804, Sigma, USA) antibodies were used in the co-immunoprecipitation assays. Total protein lysate was obtained in immunoprecipitation buffer. The total protein concentration of the supernatants was quantified using a Bio-Rad DC™ Protein Assay Reagent A/B/S (Bio-Rad, USA). 500 μg of total protein was mixed with 1 μg the primary antibody, or IgG, and the mixture were shaken on a rotating shaker at 4°C for 2 hours. Beads (Protein G Sepharose 4 Fast Flow, GE Healthcare Life Sciences, Piscataway, NJ, USA) were added to the mixture and shaken at 4°C for 1 hour. Then the beads were collected by centrifugation and washed three times by immunoprecipitation buffer. 2× sample loading buffer was added to the beads before boiling for 5 minutes. The supernatant was collected and used in the immunoblotting assays.

### Cell proliferation and cell viability assays

For the proliferation assay, HCC cells were seeded into 96-well plates at 5000 cells per well for 24 hours and assessed using a Cell Proliferation ELISA, BrdU (5-bromodeoxyuridine) (chemiluminescent) (Roche, USA). The 3-(4, 5-dimethylthiazol-2-yl) 2, 5-diphenyl tetrazolium bromide (MTT, Roche, USA) assay was used to assess cell viability at 24, 48 and 72 hours.

### Cell apoptosis detection

An Annexin-V-FLUOS Staining Kit (Roche, USA) was used to analyze the level of apoptosis, as previously described [17]. The caspase 3/7 activity assay was conducted using an Apo-ONE® Homogeneous Caspase-3/7 Assay (Promega, Madison, WI, USA), as described in our previous study [28].

### *In vivo* experiments

A nude mouse xenograft model was established using 4–6 week-old female BALB/c nude mice (Centre of Laboratory Animals, The Medical College of Xi’an Jiaotong University, Xi’an, China). Mice were housed in sterilized cages (2 animals/cage) at a constant temperature and humidity and fed a regular autoclaved chow diet with water ad libitum [17]. As HepG2 is not a tumorigenic cell line in immunosuppressed mice as described in ATCC (American Type Culture Collection), 5 × 10^6^ Hep3B cells were inoculated subcutaneously into the flank of each nude mouse. The tumor volume for each mouse was determined by measuring two of its dimensions and then calculated as tumor volume = length × width × width/2. After 3 weeks, the mice were sacrificed by cervical dislocation under anesthesia with ether and the xenograft tumor tissue was explanted for routine pathological examination. The amount of apoptosis in the isolated tumor tissues was detected using a TUNEL (Terminal-deoxynucleoitidyl Transferase Mediated Nick End Labeling) assay kit (4810-30-K, R&D Systems, Minneapolis, Minnesota, USA) according to the manufacturer’s guidelines. All animal protocols were approved by the Institutional Animal Care and Use Committee of Xi’an Jiaotong University.

### Statistical analysis

All date are presented as the Mean ± SEM. The SPSS statistical package for Windows Version 13 (SPSS, Chicago, IL, USA) was used for the Pearson chi-square tests and the multi-variant Cox regression analysis. A two-tailed Student’s *t* test, a Kaplan–Meier plot, a log-rank test, a Pearson's correlation coefficient analysis or an ANOVA was used to evaluate statistical significance using GraphPad Prism 5 software (GraphPad Software, Inc, San Diego, CA, USA). *P* < 0.05 was considered to be statistically significant.

## Results

### Clinical significance of reduced Fbxw7 expression in HCC specimens

**Figure 1:**
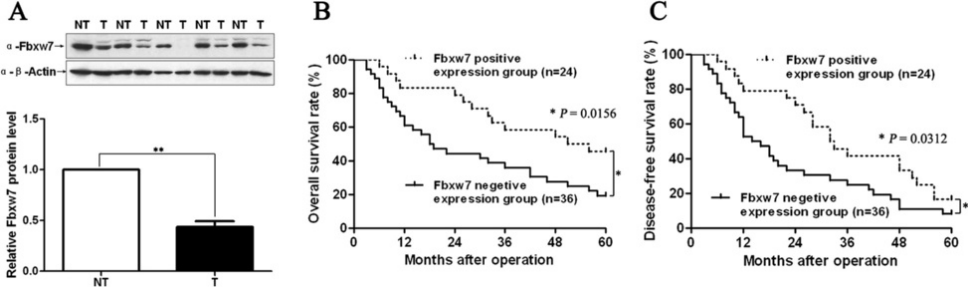
**Expression of Fbxw7 and its clinical significance in HCC cases. A)** Representative western blot analysis of Fbxw7 expression in cancer (T) and matched noncancerous tissues (NT) was shown. Quantification of the data revealed that Fbxw7 protein expression in HCC tissues was significantly lower than that in the normal tumor-adjacent tissues. n = 20; Values are depicted as Mean ± SEM; **P < 0.01 by *t* test. **B)** Kaplan–Meier overall 5-year and **C)** disease-free survival curves for HCC patients according to their Fbxw7 protein expression status. The Fbxw7 positive expression group (n = 24), IHC score of Fbxw7 = 1–3; Fbxw7 negative expression group (n = 36), IHC score of Fbxw7 = 0; *P < 0.05 by log-rank test.

To investigate the clinical significance of Fbxw7 in HCC, we tested the expression of the Fbxw7 protein by immunostaining in a retrospective cohort of 60 pairs of cancerous and matched noncancerous tissue samples from HCC patients after liver resection. Fbxw7 immunoreactivity was considered as either negative (score 0) or positive (scores 1 to 3). In these 60 cases, Fbxw7 expression was detected in 43 (71.7%) of the normal tumor-adjacent tissues, whereas only 24 (40%) of the HCC specimens showed a positive Fbxw7 signal (*P* < 0.01). Furthermore, 20 cases were subjected to immunoblotting for Fbxw7; we found that the Fbxw7 protein level in HCC tissues was significantly lower than that in the matched normal, tumor adjacent, tissues (*P* < 0.01, Figure 1A). Clinical association analysis using a Pearson chi-squared test revealed that the reduced Fbxw7 expression in HCC tissues was significantly associated with a large tumor size (≥5 cm; *P* = 0.002), venous infiltration (*P* = 0.015), high histological grade (Edmondson–Steiner grade III + IV; *P* = 0.006) and advanced tumor stage (TNM stage III + IV; *P* = 0.005) (Table 1).

### Positive Fbxw7 expression correlates with a better 5-year survival for HCC patients

A total of 60 HCC patients had complete clinical information. To determine the role of Fbxw7 in predicting the prognosis of HCC patients, immunohistostaining of Fbxw7 was performed to confirm the correlation between Fbxw7 expression and 5-year patient survival. We constructed Kaplan–Meier survival curves using the overall 5-year patient survival date to analyze cases with positive and negative Fbxw7 staining. Our date indicate that overall survival in the Fbxw7 positive expression group was 45.83%, compared with 19.44% in the negative group. In the overall survival curve, patients in the Fbxw7 negative expression group (n = 36) had a significantly poorer prognosis than those in the Fbxw7 positive expression group (n = 24; log-rank = 5.850; *P* = 0.0156; Figure 1B). The median disease-free survival times in the Fbxw7 positive and negative subgroups of HCC patients were 32.5 months and 15.0 months, respectively. Kaplan–Meier analysis also revealed that Fbxw7 loss was associated with a short disease-free survival time (log-rank = 4.643; *P* = 0.0312; Figure 1C). These data suggest that Fbxw7 may function as a potential prognostic marker in HCC. Furthermore, Fbxw7 expression is an independent factor for predicting both 5-year overall and disease-free survival in HCC patients (*P* = 0.018 and 0.007, respectively; Table 2).

**Table 2:** Multivariate Cox regression analysis of the overall and disease-free 5-year survival of 60 HCC patients

**Variables**	**Overall survival**			**Disease-free survival**		
	**HR**	**95% CI**	***P***	**HR**	**95% CI**	***P***
Age	2.113	0.888-5.030	0.091	1.670	0.787-3.545	0.182
Sex	0.749	0.339-1.652	0.474	1.031	0.492-2.162	0.936
HBV	1.846	0.694-4.908	0.219	1.971	0.827-4.696	0.126
No. of tumor nodules	1.092	0.500-2.386	0.824	0.889	0.438-1.805	0.744
Tumor size	0.411	0.160-1.056	0.065	0.481	0.211-1.098	0.082
Venous infiltration	1.539	0.722-3.284	0.264	1.892	0.940-3.808	0.074
Serum AFP level	0.883	0.412-1.895	0.749	0.953	0.483-1.880	0.889
Cirrhosis	1.288	0.651-2.548	0.468	1.313	0.711-2.423	0.384
Edmondson-Steiner grading	0.771	0.322-1.846	0.560	0.702	0.338-1.458	0.342
TNM tumor stage	3.576	1.251-10.211	0.017*	3.532	1.266-9.858	0.016*
Fbxw7 expression in tumor	0.253	0.081-0.790	0.018*	0.228	0.078-0.666	0.007*

HBV, hepatitis B virus; AFP, alpha-fetoprotein; TNM, tumor-node-metastasis; HR, hazard ratio; CI, confidence interval. *Statistically significant.

### Fbxw7 inhibits proliferation and induces apoptosis in HCC cells

**Figure 2:**
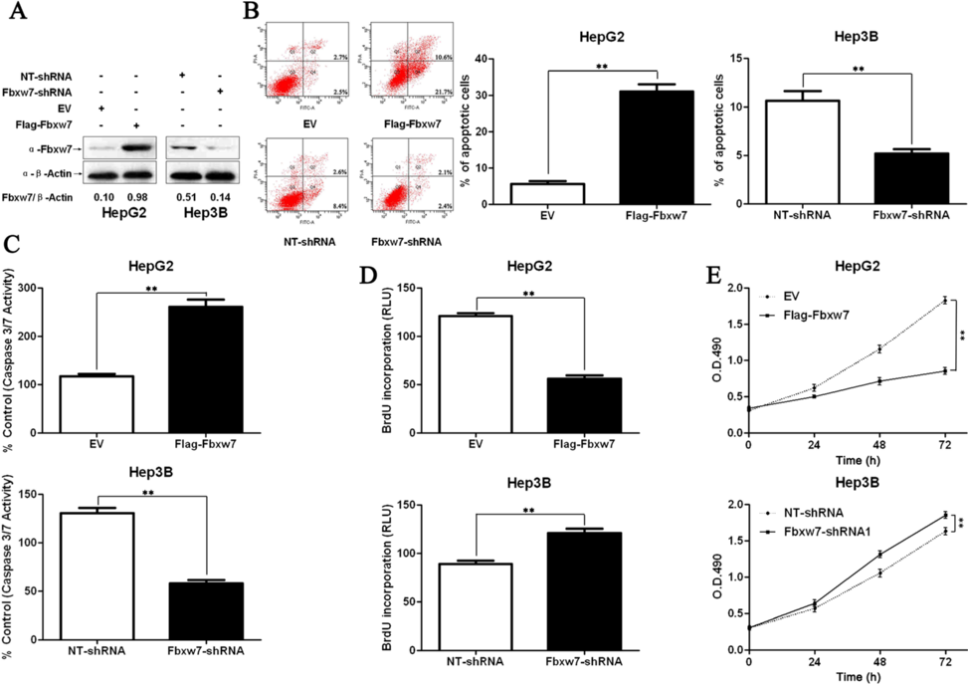
**Fbxw7 regulates apoptosis and proliferation in HCC cells. A)** HepG2 and Hep3B cells that had been transfected with Flag-Fbxw7 and Fbxw7-shRNA, respectively, were subjected to western blotting for Fbxw7. The data are representative of multiple repeats with similar results. **B)** Quantification of the apoptotic cell population by flow cytometry. Fbxw7 overexpressing HepG2 cells were composed of a larger subset of apoptotic cells compared with the control cells and Fbxw7 knockdown decreased the percentage of apoptotic Hep3B cells. **P < 0.01 by *t* test; n = 3 repeats with similar results. **C)** The activity of the pro-apoptotic caspases 3 and 7 was up-regulated after Fbxw7 overexpression in HepG2 cells and down-regulated after Fbxw7 knockdown in Hep3B cells. **P < 0.01 by *t* test; n = 3 repeats with similar results. **D)** Cell proliferation as measured by BrdU incorporation was inhibited by Fbxw7 overexpression in HepG2 cells and promoted by Fbxw7 knockdown in Hep3B cells. **P < 0.01 by *t* test; n = 3 repeats with similar results. **E)** As assessed by MTT assays, Fbxw7 overexpression was found to reduce the viability of HepG2 cells and Fbxw7 knockdown was found to enhance the viability of Hep3B cells. **P < 0.01 by two-way ANOVA; n = 3 repeats with similar results. Values are depicted as Mean ± SEM.

Our previous studies have shown that Fbxw7 knockdown in HCC cells, using a siRNA, enhanced cell viability and partially abolished p53-induced apoptosis [17]. We thus sought to determine whether Fbxw7 acted as a tumor suppressor in HCC in a similar fashion, i.e. by promoting apoptosis and inhibiting cell proliferation. As assessed by western blotting analysis, the Fbxw7 protein level could be raised by ectopically expressing a flag tagged Fbxw7 (Flag-Fbxw7) or reduced using an Fbxw7 shRNA, in HepG2 and Hep3B cells respectively (Figure 2A). As determined by flow cytometry, apoptosis assays and caspase 3/7 activity assays, Fbxw7 overexpression induced apoptosis in HepG2 cells and Fbxw7 knockdown prevented Hep3B cells from undergoing apoptosis (*P* < 0.01, respectively, Figure 2B and C). BrdU and MTT assays were performed to test the effect of altering Fbxw7 levels on tumor cell proliferation and viability, respectively. As expected, Fbxw7 overexpression inhibited proliferation and viability in HepG2 cells and Fbxw7 knockdown promoted the proliferation and viability of the Hep3B cells (*P* < 0.01, for both assays, Figure 2D and E). Hep3B has higher basal expression of Fbxw7 than HepG2. Accordingly, our data showed that HepG2 had less baseline apoptosis and more proliferation as well as viability than Hep3B (Figure 2). Thus, Fbxw7 exerts an anti-HCC effect by promoting both apoptosis and growth arrest.

### Fbxw7 inversely correlates with YAP protein in HCC tissues

**Figure 3:**
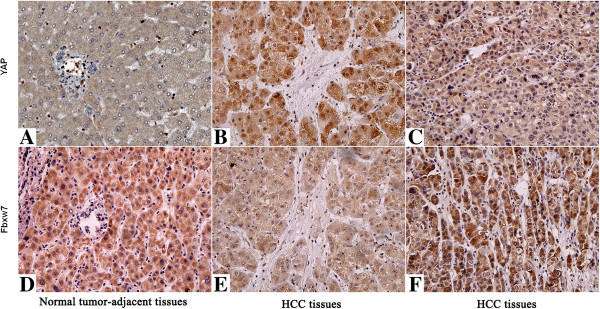
**Immunohistochemical analyses of YAP and its correlation with Fbxw7 protein in HCC.** In cases of high Fbxw7 protein expression **(D, F)**, there was no detectable YAP protein expression **(A, C)** in the same tissue section. In contrast, in the case of low Fbxw7 protein expression **(E)**, there was strong YAP protein expression **(B)**. The fibrotic septa **(B, E)** simply represent fibrotic tumor stroma with normal background liver. Scale bar: 100 μm.

Since YAP overexpression has been reported in HCC [25], we examined the correlation between Fbxw7 and YAP in serial sections of 60 HCC cases by immunohistochemical study. YAP immunoreactivity was considered as either negative (score 0) or positive (scores 1 to 3). The expression of YAP protein in cancer tissues was significantly higher than those in paired noncancerous tissues [63.3% (38/60) *vs* 11.7% (7/60); *P* < 0.01]. Furthermore, IHC scores were used for semiquantitative analysis of Fbxw7 and YAP expression, we found a strong inverse correlation between Fbxw7 and YAP in HCC tissues (*r* = -0.572; *P* = 0.003; Figure 3).

### Fbxw7 regulates YAP abundance in HCC cells

**Figure 4:**
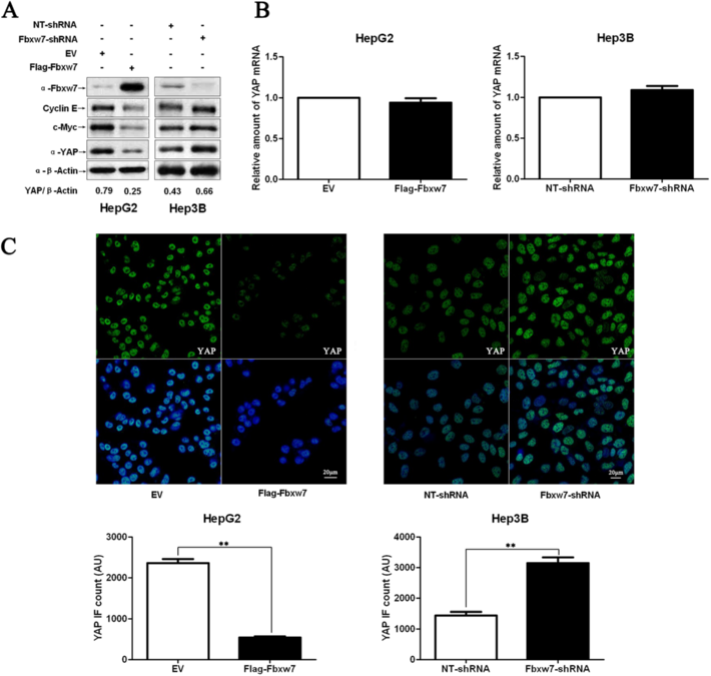
**Fbxw7 regulates the stability of the YAP protein in HCC cells. A)** HepG2 and Hep3B cells that were transfected with Flag-Fbxw7 and Fbxw7-shRNA, respectively, and subjected to Western blotting for Fbxw7, c-Myc, Cyclin E and YAP. Fbxw7 overexpression decreased c-Myc, Cyclin E and YAP protein levels in HepG2 cells, whereas Fbxw7 knockdown led to c-Myc, Cyclin E and YAP accumulation in Hep3B cells. Data are representative of multiple repeats with similar results. **B)** HepG2 cells transfected with EV or Flag-Fbxw7 and Hep3B cells transfected with non-targeting (NT)-shRNA or Fbxw7-shRNA were harvested for RNA extraction and real time RT-PCR. Fbxw7 overexpression or knockdown did not change *YAP* mRNA levels. n = 3 independent experiment; Values are depicted as the Mean ± SEM. **C)** HCC cells that were treated as above were subjected to IF for YAP. Quantification of YAP immunofluorescence revealed that the average level of YAP in the control cells was significantly higher than that in the Fbxw7 overexpressing HepG2 cells and lower than that in the Fbxw7 shRNA transfected Hep3B cells. Scale bar: 20 μm, n = 6; Values are depicted as the Mean ± SEM; **P < 0.01 by *t* test.

To investigate the downstream factor(s) involved in Fbxw7-mediated apoptosis and growth arrest in HCC; HepG2 and Hep3B cells were transfected with Flag-Fbxw7 and Fbxw7-shRNA respectively. Western blotting analysis was performed to detect c-Myc, Cyclin E and YAP. c-Myc and Cyclin E are both confirmed target proteins of Fbxw7. YAP functions as a transcriptional coactivator involved in the regulation of cell growth, proliferation, and apoptosis [19]. Fbxw7 knockdown led to c-Myc and Cyclin E accumulation in Hep3B cells and Fbxw7 overexpression decreased the levels of both proteins in HepG2 cells (Figure 4A). These results are consistent with those of our prior study [17]. Interestingly, we found that Fbxw7 overexpression also decreased YAP protein levels in HepG2 cells and that Fbxw7 knockdown increased the level of this protein in Hep3B cells (Figure 4A), whereas YAP mRNA levels were only either slightly decreased or slightly increased, without statistically significant (Figure 4B). Furthermore, IF for YAP indicated that the average level of YAP in Flag-Fbxw7 transfected HepG2 cells was significantly lower than that in control cells (*P* < 0.01, Figure 4C). Conversely, the average level of YAP in Fbxw7-shRNA transfected Hep3B cells was significantly higher than that in control cells (*P* < 0.01, Figure 4C). These data indicate that Fbxw7 reduces YAP protein levels in HCC cells.

### Fbxw7 promotes the ubiquitination and proteasomal degradation of YAP

**Figure 5:**
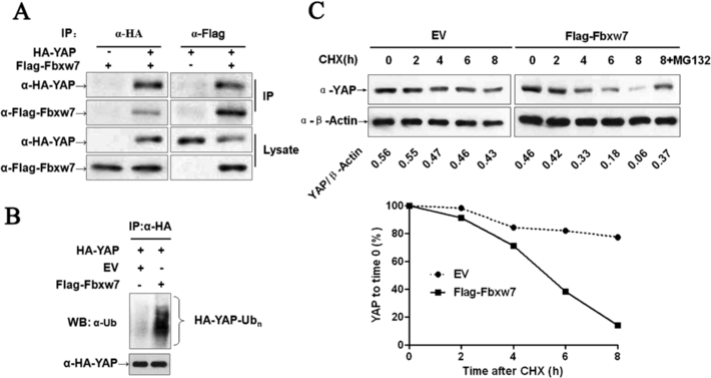
**Fbxw7 binds to YAP and promotes the ubiquitin-mediated proteolysis of YAP. A)** YAP and Fbxw7 coimmunoprecipitate with each other. Flag-Fbxw7 and HA-YAP plasmids were transfected into HEK293 cells. YAP or Fbxw7 was immunoprecipitated with anti-HA or anti-Flag antibody. Western blotting was performed to detect the specific proteins indicated on the left side of each panel. **B)** HA-YAP was precipitated from HA-YAP overexpressing HepG2 cells by immunoprecipitation using an anti-HA antibody; YAP ubiquitination was detected by western blotting. Fbxw7 overexpression markedly promoted YAP ubiquitination. **C)** The Fbxw7 turnover rate was shorter in Fbxw7 overexpressing HepG2 cells. The protein half-life of YAP was analyzed following treatment with cycloheximide. The YAP band intensity was normalized to GAPDH and then normalized to t = 100 controls. The half-life of YAP in Flag-Fbxw7 = 5.2 h (R^2^ = 0.96) and in EV = 16.5 h (R^2^ = 0.92). Additionally, treatment with MG132 (a proteasome inhibitor) inhibited Fbxw7 induced YAP degradation in HepG2 cells. The data are representative of multiple independent experiments.

Because Fbxw7 is an ubiquitin ligase that targets several oncoproteins for proteolysis and YAP is known to be subjected to ubiquitin modification by β-TrCP [1,26], we sought to determine whether Fbxw7 binds to YAP and leads to its ubiquitination and proteasomal degradation. To this end, the interaction between Fbxw7 and YAP was confirmed in HEK293 cells by reciprocal co-IP of HA-YAP and Flag-Fbxw7 (Figure 5A). HA-YAP was precipitated from HepG2 cells expressing HA-YAP, and YAP ubiquitination was detected by an ubiquitin western blot. As shown in Figure 5B, Fbxw7 overexpression markedly increased the ubiquitination of YAP in HepG2 cells. We next used a CHX (Cycloheximide, a protein synthesis inhibitor) chase assays to analyze the Fbxw7 mediated downregulation of YAP in both control and Fbxw7 overexpression HepG2 cells. To this end, the half-life of YAP was substantially decreased in Fbxw7-overexpressing cells, compared with that seen in the control cells (5.2 h *vs* 16.5 h, Figure 5C). Additionally, the proteasome inhibitor MG132 was able to prevent the downregulation of YAP (Figure 5C). Thus, these data support a model whereby Fbxw7 binds to YAP and thereby promotes the ubiquitination and proteasomal degradation of YAP.

### Fbxw7 inhibits proliferation and induces apoptosis through promoting YAP degradation in HCC

**Figure 6:**
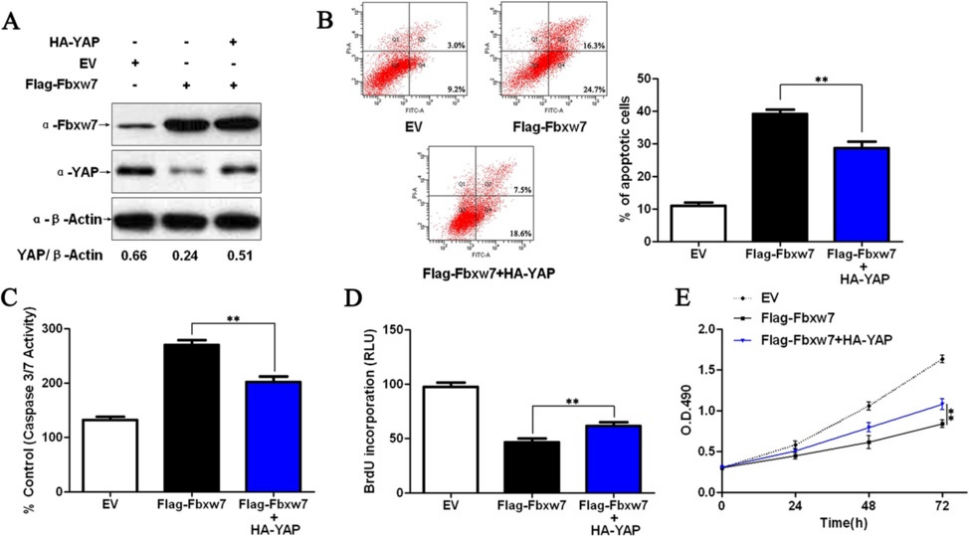
**Fbxw7’s suppression of Hep3B cell growth was partially reverted by YAP. A)** Flag-Fbxw7 transfected Hep3B cells successfully up-regulated Fbxw7 protein expression as shown by western blot. Fbxw7 overexpression in the same cell line could reduce the levels of YAP. Fbxw7 over-expressing cells that were transfected with HA-YAP partially rescued the phenotype, showing higher YAP levels. The data are representative of multiple repeats with similar results. **B)** Apoptotic cells were measured by flow cytometry. Restoring YAP expression decreased the percentage of apoptotic cells in Flag-Fbxw7 transfected Hep3B cells. **P < 0.01 by one-way ANOVA; n = 3 repeats with similar results. **C)** The activity of the pro-apoptotic caspases 3 and 7 in Fbxw7 overexpressing Hep3B cells was decreased by HA-YAP transfection. **P < 0.01 by one-way ANOVA; n = 3 repeats with similar results. **D)** A BrdU assay showed that YAP promotes proliferation in Fbxw7 overexpressing Hep3B cells. **P < 0.01 by one-way ANOVA; n = 3 repeats with similar results. **E)** YAP was found to enhance the viability of Fbxw7 over-expressing Hep3B cells (MTT assay). **P < 0.01 by two-way ANOVA; n = 3 repeats with similar results. Values are depicted as the Mean ± SEM.

**Figure 7:**
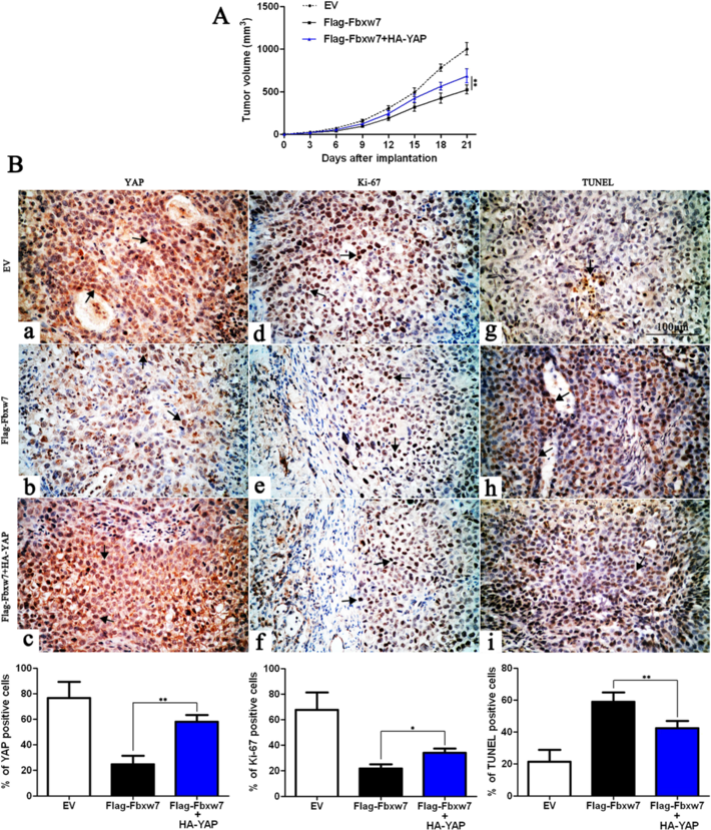
**YAP partially abolishes Fbxw7’s suppression of tumor growth. A)** Control Hep3B cells (EV, n = 6), Fbxw7 overexpressing Hep3B cells (Flag-Fbxw7, n = 6) and co-expressing Hep3B cells (Flag-Fbxw7 + HA-YAP, n = 6), respectively, were implanted into nude mice via subcutaneous injection. Tumor nodules were measured using a caliper at different times after implantation. Fbxw7 overexpressing Hep3B cells exhibited a greater tumor-inhibiting effect compared with control cells; however, restoring YAP expression accelerated tumor growth, compared with the Flag-Fbxw7 group. **P < 0.01 by two-way ANOVA. **B)** Tumor nodules were subjected to immunohistochemical staining for YAP and Ki-67, TUNEL assays and quantitative analysis. Representative immunostaining and TUNEL assays revealed that Fbxw7 overexpression significantly reduced the number of YAP and Ki-67 positive cells and increased the number of apoptotic cells. However, the percentage of YAP and Ki-67 positive cells in tumors arising from the Flag-Fbxw7 + HA-YAP group was significantly higher than that in the tumors from the Flag-Fbxw7 group and the percentage of apoptotic cells in the Flag-Fbxw7 + HA-YAP group was significantly lower than that in the Flag-Fbxw7 group. Black arrows indicate positive cells in each photomicrograph. Scale bar: 100 μm; n = 6; Values are depicted as the Mean ± SEM; *P < 0.05 and **P < 0.01 by one-way ANOVA.

To determine whether the YAP protein participates in Fbxw7 induced apoptosis and growth arrest in HCC cells, Fbxw7 overexpressing Hep3B cells were subsequently transfected with HA-YAP. Restoring YAP expression in Hep3B cells partially reverted the effect of exogenous Fbxw7 overexpression, leading to a significant reduction in the number of apoptotic cells as well as increased cell proliferation and viability (*P* < 0.01, respectively, Figure 6). We next sought to determine whether Fbxw7 affects tumor growth by inhibiting YAP using a Hep3B subcutaneous tumor model. Hep3B cells that had been infected with different retroviruses were implanted into nude mice via subcutaneous injection. Tumor growth curves, generated over 21 days, revealed that Fbxw7 overexpression slowed down Hep3B tumor growth in mice. Restoring YAP expression partially restored tumor growth, for the Fbxw7-overexpressing Hep3B cells (*P* < 0.01, Figure 7A). We performed immunohistochemistry for YAP and Ki-67 as well as TUNEL assays in the xenografted tissues. As expected, Fbxw7 overexpression down-regulated YAP protein expression, inhibited proliferation and induced apoptosis *in vivo*. YAP partially abolished the inhibiting effect of Fbxw7 on HCC growth; it led to a significant reduction in the number of apoptotic cells and increased the number of cells staining positive for Ki-67, which is consistent with our *in vitro* observations (*P* < 0.05 or *P* < 0.01, respectively, Figure 7B). Taken together, these data indicate that YAP may function as a downstream factor in Fbxw7 induced apoptosis and growth arrest in HCC.

## Discussion

As a general tumor suppressor, both Fbxw7 mRNA and protein expression levels have been shown to be down-regulated in various cancers [10]. Additionally, dysfunctional mutations of Fbxw7 have been found in several malignancies and the total mutation rate was reported as 6% [11]. We initially investigated the expression of the Fbxw7 protein in 60 HCC patients using immunohistochemistry and western blotting, and our data showed that the expression of Fbxw7 was significantly lower in HCC compared with matched normal tumor-adjacent tissues. Furthermore, Fbxw7 expression was significantly correlated with tumor size, venous infiltration, Edmondson–Steiner grading and TNM tumor stage, which is consistent with our previous study [15,16]. Importantly, our date indicates that Fbxw7 positive expression is significantly correlated with a better 5-year patient survival for all HCC patients, which is consistent with the previous studies on gastric cancer, colorectal cancer, glioma and melanoma [12,14,29,30]. Otherwise, multivariate Cox repression analysis found that Fbxw7 is an independent factor in predicting both overall 5-year survival and disease-free survival in HCC patients. These results show that the status of Fbxw7 is critical for prognosis determination in HCC patients. Our previous study showed that Fbxw7 functions as a tumor suppressor and that it may be involved in apoptosis and proliferation in HCC [17]. In this study, we have further confirmed that Fbxw7 suppresses tumor progression by promoting apoptosis and growth inhibition in HCC.

It has been reported that Fbxw7 is responsible for the degradation of several substrates, which are involved in apoptosis and cell proliferation regulation, such as c-Myc, Cyclin E, Notch1 and MCL-1 [2,3,7,8]. Our previous study reported that Fbxw7 levels were inversely correlated with those of c-Myc and Cyclin E in HCC tissues [15]. Additionally, Fbxw7 knockdown by siRNA led to an accumulation of c-Myc and Cyclin E in HCC cells [17]. In this study, we confirmed that YAP expression in HCC was obviously higher as compared with those in normal tumor-adjacent tissues. Otherwise, Fbxw7 was inversely associated with YAP protein expression in HCC tissues. *In vitro* studies, we found that Fbxw7 negatively regulates c-Myc and Cyclin E abundance in HCC cells. Interestingly, we found that Fbxw7 overexpression significantly decreased YAP levels in HepG2 cells. Conversely, the ablation of Fbxw7 by shRNA in Hep3B, a cell line with high expression of the target gene, consistently showed an accumulation of YAP. But YAP mRNA levels did not change significantly with Fbxw7 regulation, suggesting that Fbxw7 regulates the abundance of the YAP protein in HCC cells. It has been reported that the β-TrCP E3 ubiquitin ligase catalyzed YAP ubiquitination, ultimately leading to YAP degradation [26]. In our study, we confirmed the interaction between Fbxw7 and YAP in HEK293 cells using co-immunoprecipitation. Furthermore, co-IP and western blot indicated that Fbxw7 promotes YAP ubiquitination and shortens the half-life of YAP in HepG2 cells, and that MG132 treatment could inhibit YAP’s downregulation by Fbxw7. The data we present in this study indicate that Fbxw7 mediates the ubiquitination of YAP, and thus its subsequent proteasomal degradation. In most cases, phosphorylation of the F-box recognition motif present on the substrate, more commonly known as a phosphor-degron, is necessary before efficient substrate/ligase interaction [31]. Therefore, further investigation is required to confirm whether YAP is a *bona fide* target of Fbxw7 and a proteomic data with searches for YAP with the Fbxw7 canonical phospho-degron may address this question.

Here we showed Fbxw7 overexpression using a retrovirus, which resulted in the downregulation of YAP *in vitro* and *in vivo*. This and the observed increase in apoptosis and growth arrest could be partially reverted by treatment by retrovirus-mediated YAP overexpression. However, only 30%–35% of the effect of Fbxw7 on inhibiting HCC cells was abolished, despite achieving more than a 70% restoration in the levels of YAP, suggesting that YAP is not the unique downstream effector of Fbxw7 induced apoptosis and growth arrest in HCC. Fbxw7 targets various oncoproteins for ubiquitination and degradation and these substrates play important roles in regulating apoptosis, cell cycle, cell division, cell proliferation [1]. We suggest that Fbxw7 triggers apoptosis and growth arrest likely through affecting several downstream targets. Nevertheless, Fbxw7 mediated YAP degradation might account for apoptosis and growth arrest in HCC.

**Figure 8:**
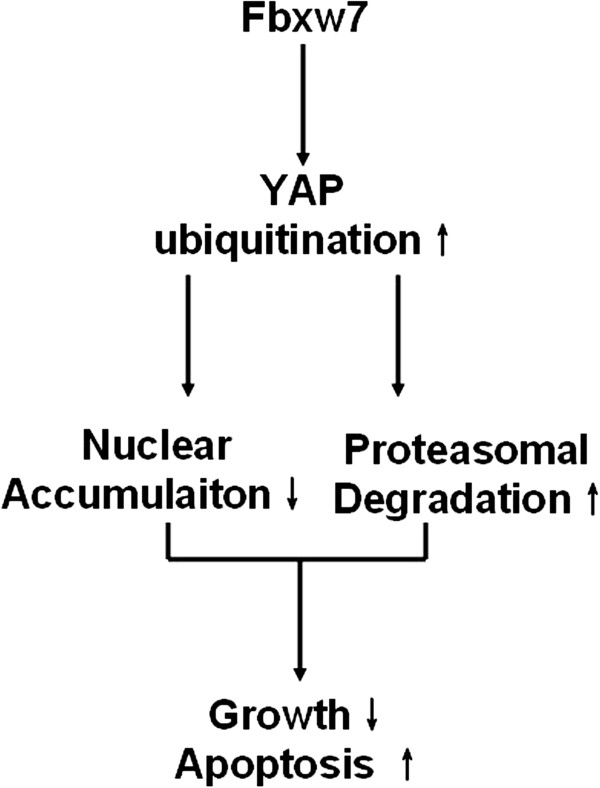
**Working model for the tumor suppressive function of Fbxw7 and its downstream pathway.** Fbxw7 promotes both apoptosis and the growth arrest of HCC through promoting YAP ubiquitination and proteasomal degradation.

In conclusion, we demonstrate that reduced levels of Fbxw7 are associated with poor clinicopathological characteristics in HCC tissues. The negative expression of Fbxw7 is an independent factor for predicting poor prognosis in HCC patients. Fbxw7 induces apoptosis and growth arrest by promoting the ubiquitination and proteasomal degradation of the YAP protein, both *in vitro* and *in vivo* (Figure 8). Altogether, we hypothesize that loss of Fbxw7 function contributes to hepatocarcinogenesis, in part through the accumulation of YAP. By addressing this pathway, we identified Fbxw7 as a potential therapeutic target for HCC.

## Conclusions

In summary, this study shows that Fbxw7 expression is impaired in cancer tissues as compared with noncancerous tissues and that reduced Fbxw7 levels are correlated with poor clinicopathological features in HCC. Furthermore, we demonstrate that Fbxw7 is an independent factor for predicting the overall 5-year survival and disease-free survival of HCC patients. *In vitro* studies found that Fbxw7 inhibits HCC growth via inducing apoptosis and growth arrest. Elevated YAP expression is observed in HCC tissues and its expression is inversely associated with Fbxw7. Importantly, our data, for the first time, indicate that Fbxw7 inversely regulates YAP protein abundance by promoting YAP for ubiquitination and proteasomal degradation in HCC. Our results confirm that YAP is a candidate oncogene because it plays a critical role in hepatocarcinogenesis. Interestingly, restoring YAP can partially abolish the effect of Fbxw7 on anti-HCC, suggesting that Fbxw7 may exert its tumor suppressive function by regulating the stability of the YAP protein. This study reveals a potential target of Fbxw7 and supplies us with a new insight into the accumulation of YAP in HCC. Fbxw7 may potentially act as a clinical biomarker, and may also be a therapeutic target, in HCC.

## Abbreviations

Fbxw7: F-box and WD repeat domain-containing 7; HCC: Hepatocellular carcinoma; YAP: Yes-associated protein; SCF: SKP1-CUL1-F-box protein; SREBP1: Sterol regulatory element binding protein-1; CPD: Cdc4 phospho-degron; TEAD: TEA domain; CHX: Cycloheximide; co-IP: Co-immunoprecipitation; FBS: Fetal bovine serum; SP-IHC: Streptavidin peroxidase conjugate-immunohistochemistry; mTOR: Mammalian target of rapamycin; MCL-1: Myeloid cell leukemia-1; RUNX2: Runt-related transcription factor 2; DMEM: Dulbecco’s modified eagle medium; qRT-PCR: Real-time quantitative reverse transcription polymerase chain reaction; MTT: 3-(4, 5-dimethylthiazol-2-yl) 2, 5-diphenyl tetrazolium bromide; BrdU: 5-bromodeoxyuridine; TUNEL: Terminal-deoxynucleoitidyl transferase mediated nick end labelin; ATCC: American Type Culture Collection; IF: Immunofluorescence.

## Competing interest

The authors declare that they have no competing interests.

## Authors’ contributions

KST, WY, CL, XZ, ZTL and CG carried out the cell biology and molecular biology experiments, participated in the sequence alignment and drafted the manuscript. YMY and QGL participated in the design of the study and performed the statistical analysis. KST conceived of the study, and participated in its design and coordination and helped to draft the manuscript. All authors read and approved the final manuscript.
